# α-Synuclein burden amplification in Parkinson’s disease: a unified genetic, molecular, and cellular framework

**DOI:** 10.3389/fneur.2026.1924943

**Published:** 2026-07-27

**Authors:** Sachiko Noda, Nobutaka Hattori

**Affiliations:** 1 Neuron–Glia Crosstalk Center, Juntendo University Graduate School of Medicine, Tokyo, Japan; 2 Department of Neurology, Juntendo University Graduate School of Medicine, Tokyo, Japan; 3 Neurodegenerative Disorders Collaboration Laboratory, RIKEN Center for Brain Science, Wako, Japan

**Keywords:** autophagy–α-synuclein homeostasis, neurodegeneration, Parkinson’s disease, protein aggregation, SNCA, α-synuclein

## Abstract

**Introduction:**

Parkinson’s disease (PD) is a progressive neurodegenerative disorder characterized pathologically by the accumulation and propagation of α-synuclein (α-syn). Although α-syn aggregation is considered central to PD pathogenesis, increasing evidence suggests that α-syn abundance may be as important as its conformational state. Genetic studies have demonstrated an *SNCA* dosage effect, with gene duplication and triplication associated with progressively more severe familial PD phenotypes. Complementary evidence indicates that dysfunction of protein clearance pathways, particularly the autophagy–lysosome system, promotes intracellular α-syn accumulation and increases its neurotoxic potential. In this review, we propose α-syn multiplication as an integrative framework for interpreting PD pathogenesis. This concept extends beyond *SNCA* copy-number variation to encompass processes that increase the effective α-syn burden within neurons or across neural networks, including increased gene expression, impaired degradation, disrupted proteostasis, and pathological propagation.

**Methods:**

We summarize α-syn structural dynamics and the concentration-dependent distribution of monomeric, oligomeric, and fibrillar species. We then review evidence from *SNCA* gene-dosage studies and examine the role of the autophagy–lysosome pathway in regulating α-syn homeostasis, with particular emphasis on recent experimental findings demonstrating that autophagy deficiency exacerbates α-syn accumulation and neurodegeneration in human α-syn bacterial artificial chromosome transgenic mice.

**Results:**

Collectively, the available genetic, biochemical, and experimental evidence supports a model in which the balance between α-syn production and clearance influences disease progression alongside protein misfolding. The interaction between increased protein burden and impaired clearance capacity provides a unifying mechanism linking familial and sporadic forms of PD.

**Discussion:**

We propose that α-syn multiplication offers an integrative framework for understanding PD pathogenesis, provides a quantitative perspective on disease heterogeneity, and highlights therapeutic opportunities aimed at reducing α-syn burden and restoring proteostatic balance.

## 1 Introduction

Parkinson’s disease (PD) is a progressive neurodegenerative disorder characterized by motor manifestations, including bradykinesia, rigidity, and tremor, together with a broad spectrum of non-motor features. Pathologically, PD is characterized by Lewy bodies and Lewy neurites, whose principal constituent is α-synuclein (α-syn) (1). Although α-syn is widely recognized as a central mediator of PD pathogenesis, the mechanisms linking its regulation to disease onset and progression remain incompletely understood. Under physiological conditions, α-syn is predominantly localized at presynaptic terminals, where it contributes to synaptic vesicle trafficking, neurotransmitter release, and maintenance of synaptic function. Dysregulation of these physiological functions, together with abnormal accumulation and aggregation of α-syn, is thought to contribute to PD pathogenesis. Aging is the strongest known risk factor for PD and is associated with progressive impairment of cellular homeostatic mechanisms, including the protein quality-control pathways that maintain proteostasis (2). These age-related changes may promote the accumulation of pathogenic α-syn species even in individuals without known PD-associated mutations.

Traditionally, PD has been conceptualized as a protein-misfolding disorder in which native α-syn undergoes aberrant conformational changes that promote oligomerization, fibril formation, and deposition as insoluble aggregates. In this model, toxic α-syn species are often regarded as either intermediates in fibrillogenesis or off-pathway assemblies. However, a strictly linear aggregation model does not fully account for several genetic and clinical observations, particularly the association between α-syn expression levels and disease severity.

Particularly informative evidence comes from hereditary PD associated with *SNCA* copy-number variation. Individuals with *SNCA* duplication typically develop a relatively late-onset parkinsonian phenotype (3, 4), whereas *SNCA* triplication is associated with earlier onset and a more aggressive disease course (5). This gene-dosage effect indicates that α-syn-related neurotoxicity is linked to quantitative increases in protein abundance, not solely to qualitative changes in protein conformation. Thus, *SNCA* copy-number multiplication provides strong genetic evidence that α-syn expression level is an important determinant of disease severity.

Recent experimental evidence further supports the concept that increased α-syn burden and impaired cellular clearance interact to accelerate neurodegenerative processes. A previous work demonstrated that autophagy loss in dopaminergic neurons induces Lewy-like pathology and motor dysfunction in aged mice (6). More recently, an *in vivo* study used human α-syn bacterial artificial chromosome transgenic mice with dopaminergic neuron-specific autophagy deficiency induced by conditional *Atg7* deletion (7). The study showed that impaired autophagic degradation exacerbated α-syn accumulation, p62-positive pathology, nigrostriatal dopaminergic neurodegeneration, and motor dysfunction (7). These findings support an interaction between α-syn burden and intracellular degradation capacity in promoting neurodegeneration.

Together, these observations support a model in which α-syn toxicity reflects not only its propensity to misfold but also protein burden and cellular clearance capacity.

Complementing the genetic and *in vivo* evidence, biochemical studies have provided a more dynamic view of α-syn structural biology. Mechanistic studies describe α-syn as occupying a dynamic conformational landscape comprising monomeric, oligomeric, and fibrillar states (8). Within this framework, oligomeric species may constitute distinct structural entities rather than merely transient intermediates and can exhibit neurotoxic properties (8). This conformational distribution is sensitive to α-syn concentration, suggesting that protein abundance can influence the relative prevalence of potentially pathogenic species (8).

Within this framework, increased α-syn levels arising from *SNCA* copy-number multiplication or impaired proteostasis may shift the conformational distribution toward oligomeric species with greater pathogenic potential (8). This concentration-dependent relationship provides a potential mechanistic link among gene dosage, protein homeostasis, and neurotoxicity.

On this basis, we propose the term “α-synuclein multiplication” as a unifying concept that encompasses both gene-dosage–driven increases in α-syn abundance and prion-like amplification of misfolded α-syn at the protein level:

1. Gene-level multiplicationGene-level α-syn multiplication refers to increased dosage of the *SNCA* (α-synuclein) gene, such as *SNCA* duplication or triplication. These copy-number gains lead to elevated α-syn protein levels and are sufficient to cause familial Parkinson’s disease in a gene dosage–dependent manner.
2. Protein-level multiplication (self-propagation/amplification)
  Protein-level α-syn multiplication refers to the prion-like self-propagation of misfolded α-syn. Once an abnormally folded α-syn species is formed, it acts as a “seed” or template that converts native α-syn into a pathogenic conformation, thereby amplifying the pool of misfolded protein. This templated conversion promotes the formation of fibrillar aggregates and drives the development of Lewy bodies and related α-syn pathology.

Beyond intracellular accumulation, pathological α-syn species can propagate between neurons and act as self-amplifying seeds, thereby extending pathology through interconnected neural networks (9, 10). This propagation model is consistent with the characteristic spatiotemporal progression of Lewy pathology described by Braak et al. (11).

Taken together, genetic, biochemical, and *in vivo* evidence supports a multilevel hypothesis in which α-syn multiplication contributes to PD pathogenesis. At the genetic level, *SNCA* copy-number variation directly increases α-syn expression (3–5). At the cellular level, elevated α-syn burden, particularly when autophagy is impaired, promotes pathological accumulation and neuronal vulnerability in experimental models (6, 7). At the molecular level, increased α-syn concentration may shift the conformational distribution toward oligomeric species with greater pathogenic potential (8). At the network level, propagation of pathological α-syn may extend disease burden across anatomically connected regions of the nervous system (9–11). At the systems level, these processes may converge to promote progressive and widespread neurodegeneration.

**Figure 1:**
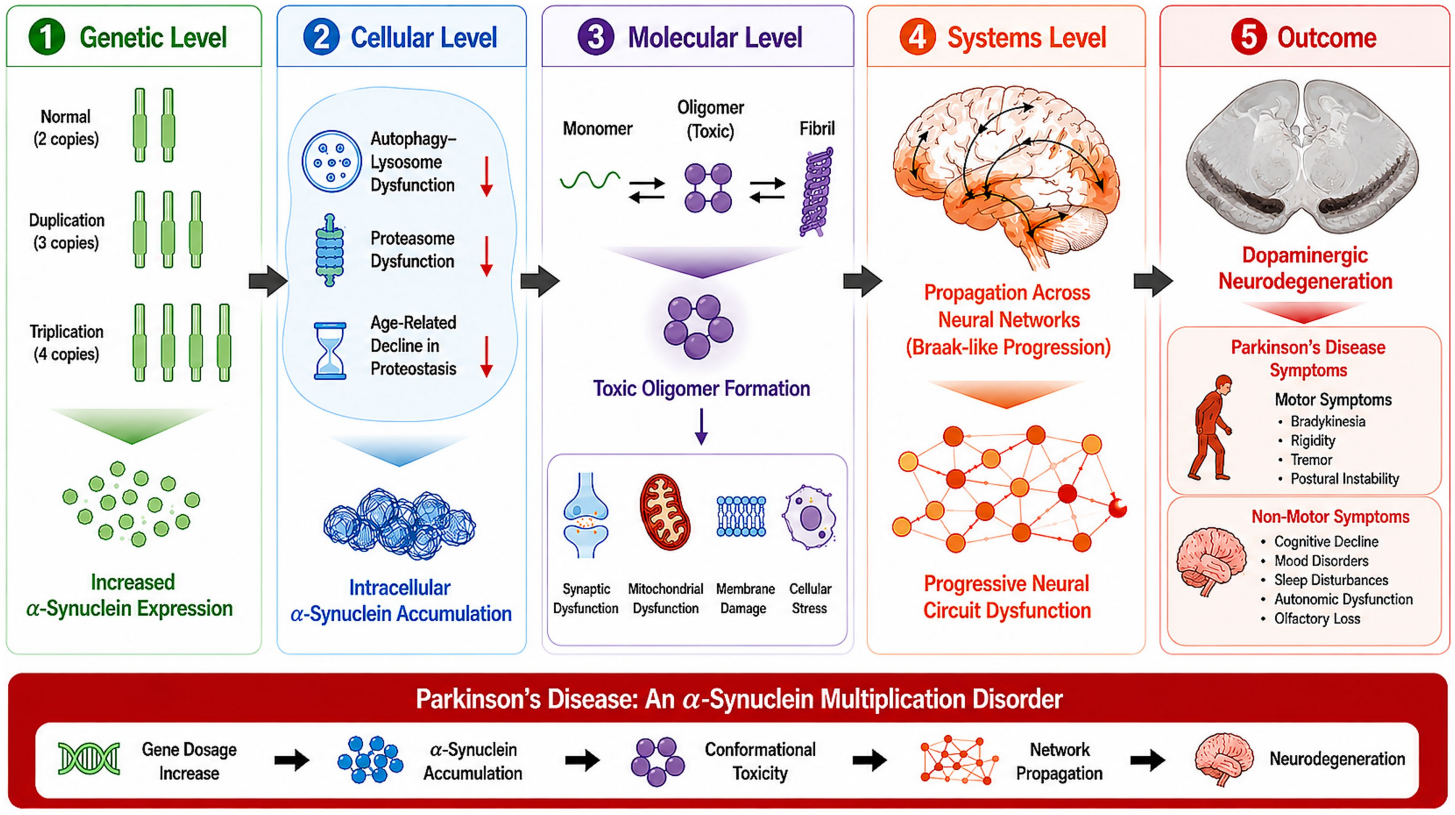
Multilevel α-synuclein multiplication in Parkinson’s disease: From gene dosage to network-level neurodegeneration. This figure illustrates the proposed α-synuclein multiplication model in Parkinson’s disease (PD). At the genetic level, *SNCA* duplication and triplication increase α-synuclein (α-syn) expression in a gene-dosage-dependent manner, thereby increasing intracellular α-syn burden. At the cellular level, impaired protein quality-control mechanisms, including autophagy–lysosome dysfunction and age-related proteostatic decline, may reduce α-syn clearance and promote intracellular accumulation. At the molecular level, increased α-syn concentration may shift the conformational distribution among monomeric, oligomeric, and fibrillar species toward oligomeric assemblies with pathogenic potential. These oligomeric species may contribute to synaptic dysfunction, mitochondrial impairment, membrane disruption, and cellular stress. At the systems level, progressive accumulation of pathogenic α-syn species may contribute to regional neuronal dysfunction, propagation of pathology across interconnected neural networks, and Braak-like disease progression. The convergence of these genetic, cellular, molecular, and systems-level mechanisms may promote dopaminergic neurodegeneration and the development of motor and non-motor manifestations of PD. Collectively, this framework conceptualizes PD as a disorder characterized by α-synuclein multiplication, in which progressive increases in α-syn burden may promote proteostatic failure, pathogenic conformational change, network-level pathology, and neurodegeneration, thereby offering a potential mechanistic link between familial and sporadic disease. α-syn, α-synuclein; PD, Parkinson’s disease; *SNCA*, α-synuclein gene. Alt text: Schematic of the proposed multilevel α-syn multiplication framework in Parkinson’s disease. Increased *SNCA* gene dosage raises α-syn expression, while impaired autophagy, lysosomal function, proteasomal activity, and age-related proteostasis reduce protein clearance. Increased intracellular α-syn promotes pathogenic oligomer formation, cellular dysfunction, network-level propagation, dopaminergic neurodegeneration, and motor and non-motor manifestations.

Despite substantial advances, gene-dosage effects, protein homeostasis, and α-syn structural dynamics are often considered separately rather than within a single integrated framework. Furthermore, age-related impairment of proteostatic and degradative pathways may increase the effective α-syn burden in individuals without pathogenic *SNCA* variants, suggesting that analogous burden-amplifying mechanisms may contribute to sporadic PD (2). This review addresses this gap by proposing α-syn multiplication as an integrative model that links genetic variation, protein abundance, clearance capacity, conformational dynamics, and pathological propagation across both familial and sporadic PD (Figure 1).

## 2 The concept of α-syn multiplication in PD

### 2.1 Gene-level multiplication

#### 2.1.1 *SNCA* gene dosage and familial PD

Strong genetic evidence for a dosage-dependent mechanism in PD comes from *SNCA* copy-number variants (3–5). *SNCA* encodes α-syn, and duplication or triplication of the gene is associated with autosomal dominant PD of differing clinical severity (3–5). This provides one of the clearest demonstrations that α-syn-related neurodegeneration is influenced by protein expression level (3–5).

Patients with *SNCA* duplication typically present with a phenotype resembling sporadic PD, including relatively late disease onset and moderate progression (3, 4). In contrast, *SNCA* triplication is associated with a more severe phenotype characterized by earlier onset, more rapid progression, and broader motor and cognitive involvement (5). Disease severity correlates with gene dosage, supporting a quantitative relationship between α-syn abundance and neurotoxicity (3–5) (Table 1).

**Table 1:** Proposed relationships between genes associated with hereditary Parkinson’s disease and α-synuclein burden.

Gene	Encoded protein	Major cellular function	Proposed relationship with α-synuclein burden
*SNCA*	α-synuclein	Synaptic vesicle regulation	Gene duplication or triplication directly increases α-syn expression and intracellular α-syn burden (3–5)
*PRKN* (*PARK2*)	Parkin	Ubiquitin-mediated proteostasis and mitophagy	Impaired clearance of damaged mitochondria may increase cellular stress and promote α-syn accumulation (28, 29)
*PINK1*	PTEN-induced kinase 1	Mitochondrial quality control and mitophagy	Defective mitophagy may increase oxidative stress and facilitate α-syn aggregation (28, 29)
*PARK7* (DJ-1)	DJ-1	Oxidative-stress response	Reduced protection against oxidative damage may promote α-syn misfolding and toxicity (28, 29)
*ATP13A2*	Lysosomal P-type ATPase	Lysosomal homeostasis	Lysosomal dysfunction may impair α-syn degradation and promote intracellular accumulation (28, 29)
*GBA1*	Glucocerebrosidase	Lysosomal sphingolipid metabolism	Reduced glucocerebrosidase activity may impair lysosomal α-syn clearance and promote aggregation (30, 31)
*VPS35*	Retromer complex component	Endosomal trafficking	Defective endosomal–lysosomal trafficking may reduce α-syn turnover (28, 29)
*LRRK2*	Leucine-rich repeat kinase 2	Vesicular trafficking and autophagy regulation	Dysregulated vesicular trafficking and autophagy may indirectly increase α-syn burden (28, 29)
*FBXO7*	F-box only protein 7	Protein quality control and mitophagy	Impaired proteostasis may promote α-syn accumulation (28, 29)
*DNAJC6*	Auxilin	Synaptic vesicle endocytosis	Abnormal vesicle recycling may alter α-syn trafficking and intracellular handling (28, 29)
*SYNJ1*	Synaptojanin-1	Synaptic vesicle recycling	Defective membrane trafficking may facilitate α-syn accumulation (28, 29)
*VPS13C*	Vacuolar protein sorting-associated protein 13C	Lysosomal and mitochondrial homeostasis	Impaired organelle quality control may promote α-syn pathology (28, 29)

α-syn; α-synuclein.

At the molecular level, *SNCA* copy-number multiplication increases transcriptional output and intracellular α-syn levels (3–5). This increase in protein abundance may perturb cellular proteostasis and promote the accumulation of pathogenic α-syn species (3–5, 8, 12, 13). Although demonstrated primarily in rare familial cases, this dosage effect may also inform our understanding of sporadic PD, in which modest increases in α-syn expression or reduced clearance capacity could produce analogous downstream effects over time (2, 6, 7). Genome-wide association studies have identified common genetic variation at the *SNCA* locus as a risk factor for sporadic PD, supporting the concept that even modest alterations in α-syn expression may contribute to disease susceptibility (14, 15).

The gene-dosage effect highlights the distinction between qualitative and quantitative models of PD pathogenesis. Whereas point mutations in *SNCA* or other PD-related genes may alter protein properties, copy-number variants directly modulate protein abundance (3–5). This observation suggests that α-syn toxicity may be sensitive to concentration thresholds beyond which cellular homeostatic mechanisms become insufficient (8).

Taken together, these observations support genetic α-syn multiplication as an important upstream contributor to disease severity in familial PD (3–5).

### 2.2 Protein-level multiplication

#### 2.2.1 Protein clearance systems: autophagy and lysosomal degradation

Intracellular protein quality-control systems, particularly the autophagy–lysosome pathway, are central to maintaining α-syn homeostasis (6, 7, 16–20). As α-syn is continuously synthesized and can misfold under physiological conditions, efficient degradation is required to limit its accumulation (6, 7). α-Syn is degraded through both autophagic and proteasomal pathways, underscoring the importance of intracellular degradation systems in maintaining protein homeostasis (18). Among these systems, macroautophagy and lysosomal degradation are the major routes for clearing soluble oligomeric and aggregated α-syn species (6, 7). Chaperone-mediated autophagy contributes to α-syn turnover, and its impairment has been implicated in α-syn accumulation and PD pathogenesis (21).

Autophagic flux is tightly regulated in neurons, which are particularly vulnerable to proteostatic stress because of their post-mitotic state and high metabolic demand. Impairment of autophagy or lysosomal function promotes the progressive accumulation of misfolded proteins and has been widely implicated in neurodegenerative disorders (16–18). In experimental models of PD, disruption of autophagy promotes presynaptic accumulation of α-syn and dopaminergic neurite degeneration, supporting a pathogenic interaction between impaired protein clearance and α-syn pathology (22). In PD, reduced clearance capacity may facilitate intracellular α-syn accumulation and increase the burden of pathogenic species (6, 7, 22). Defects in key autophagy-related genes, such as *Atg7* in mouse models, result in the accumulation of ubiquitin- and p62-positive inclusions, reflecting impaired protein degradation and cargo clearance (6).

Recent *in vivo* evidence further supports an interaction between α-syn burden and autophagy impairment in promoting neurodegeneration (7). The human α-syn bacterial artificial chromosome transgenic mouse line used in these studies was originally established as a model of behavioral abnormalities associated with α-syn overexpression (19). In human α-syn bacterial artificial chromosome transgenic mice with dopaminergic neuron-specific conditional *Atg7* deletion, autophagy deficiency exacerbated α-syn accumulation, p62-positive pathology, and nigrostriatal dopaminergic neurodegeneration (7). These pathological changes were accompanied by motor deficits, indicating that impaired proteostasis can amplify the effects of increased α-syn burden *in vivo* (7). These findings support the concept that α-syn toxicity is influenced by both protein abundance and the efficiency of intracellular clearance systems (7).

Mechanistically, the autophagy–lysosome system may provide a proteostatic reserve that maintains α-syn within a tolerable concentration range (6, 7). When this system is compromised, even normally tolerated α-syn levels may become pathogenic because of reduced clearance capacity (6, 7). Excess α-syn can itself impair macroautophagy, creating a positive-feedback loop that may accelerate intracellular protein accumulation (20). Conversely, when α-syn expression is elevated, as in *SNCA* copy-number multiplication, degradation pathways may become saturated, promoting intracellular accumulation and the formation of pathogenic species (3–5, 8). Lewy body-like α-syn aggregates may resist degradation and further disrupt autophagic function, thereby exacerbating proteostatic failure (21).

**Figure 2:**
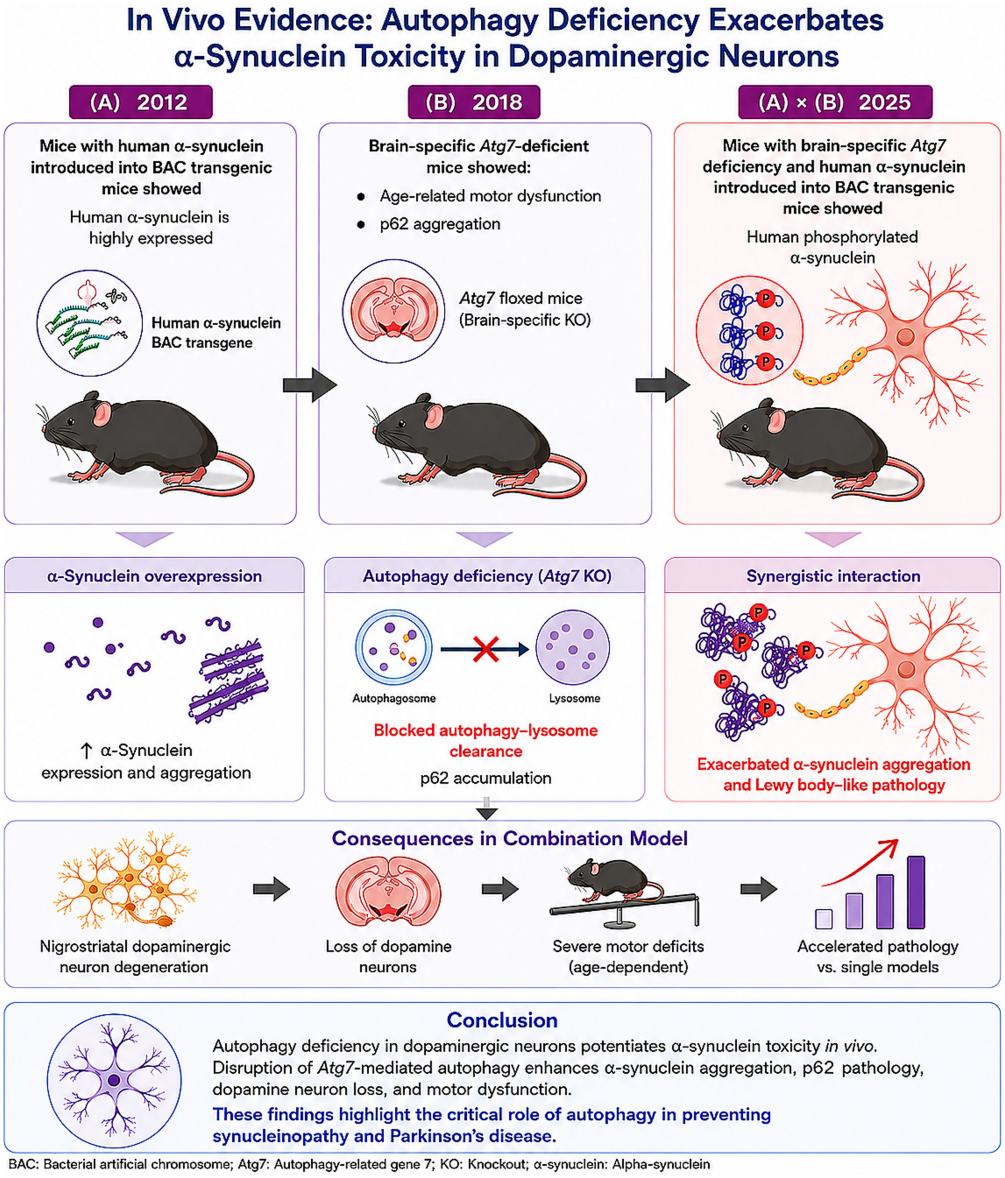
*In vivo* evidence that autophagy deficiency amplifies α-synuclein-associated pathology in dopaminergic neurons. This schematic summarizes experimental findings supporting an interaction between α-synuclein (α-syn) burden and autophagy deficiency in Parkinson’s disease–related pathology. Human α-synuclein bacterial artificial chromosome (BAC) transgenic mice exhibit increased α-syn expression, whereas mice with dopaminergic neuron-specific *Atg7* deficiency show impaired autophagy–lysosome function, p62 accumulation, and age-related motor dysfunction. To investigate the combined effects of these mechanisms, a double-mutant model was generated by crossing human α-syn BAC transgenic mice with mice carrying dopaminergic neuron-specific conditional *Atg7* deletion. The combination of elevated α-syn expression and impaired autophagic clearance was associated with greater α-syn aggregation, increased phosphorylated α-syn pathology, accelerated nigrostriatal dopaminergic neurodegeneration, and severe age-dependent motor deficits. These findings indicate that autophagy deficiency amplifies α-syn-associated pathology *in vivo* and support the concept that intracellular protein-clearance capacity helps determine the threshold at which α-syn transitions from a physiological protein to a pathogenic species.

Thus, the autophagy–lysosome pathway should be considered not merely a downstream component of neurodegeneration but also a key regulator of the threshold at which α-syn transitions from a physiological protein to a pathogenic species (6, 7). The balance between α-syn synthesis and degradation therefore represents a critical regulatory axis in PD pathogenesis (3–8) (Figure 2).

### 2.3 Α-Syn structure and aggregation dynamics

α-syn is an intrinsically disordered protein that lacks a stable secondary or tertiary structure under physiological conditions (8, 12). This structural plasticity enables α-syn to adopt multiple conformational states influenced by environmental factors, such as pH, lipid interactions, metal ions, and protein concentration (8, 12). Rather than adopting a single native conformation, α-syn exists as a dynamic ensemble of interconverting structural species (8). α-syn Structure and Aggregation Dynamics: Environmental factors, including pesticides, heavy metals, and other neurotoxins, may further promote α-syn accumulation and aggregation by impairing proteostasis, inducing oxidative stress, and disrupting mitochondrial function. These observations support the concept that genetic and environmental factors converge to increase the pathogenic burden of α-syn in PD (23).

In solution, α-syn exists predominantly as a monomer but can self-associate to form soluble oligomeric assemblies and, under certain conditions, insoluble fibrillar aggregates (8). Increasing evidence suggests that these states do not form a simple linear pathway from monomer to fibril. Instead, α-syn aggregation may be better described as a dynamic conformational landscape in which oligomeric species form stable on-pathway or off-pathway assemblies with distinct biochemical and biophysical properties (8). Among these species, soluble oligomers have attracted particular attention because of their neurotoxic potential (8, 13). Oligomeric α-syn species reportedly disrupt synaptic function, impair membrane integrity, induce mitochondrial dysfunction, and trigger inflammatory responses (8, 13). These effects can occur in the absence of mature fibril formation, suggesting that α-syn toxicity is not strictly dependent on end-stage aggregation (8).

Recent experimental and structural studies further support the concept that α-syn occupies a concentration-dependent conformational landscape (8). In this model, the relative distribution of monomeric, oligomeric, and fibrillar species is governed by thermodynamic and kinetic factors that are sensitive to α-syn abundance (8). Consequently, even modest increases in α-syn concentration may shift the conformational distribution toward oligomer formation, thereby increasing cellular toxicity (8, 12, 13).

This dynamic view of α-syn structure provides a mechanistic basis for understanding how changes in protein abundance—such as those arising from *SNCA* copy-number multiplication (3–5) or impaired degradation (6, 7)—may influence disease-associated conformational states. Thus, α-syn aggregation may be understood not only as a qualitative misfolding event but also as a quantitative, concentration-dependent process (8) (Figure 1). Environmental factors, including pesticides, heavy metals, and other neurotoxins, may further promote α-syn accumulation and aggregation by impairing proteostasis, inducing oxidative stress, and disrupting mitochondrial function. These observations support the concept that genetic and environmental factors converge to increase the pathogenic burden of α-syn in PD.

### 2.4 Α-Syn multiplication model in PD

In this framework, we propose that PD can be conceptualized as a disorder influenced by α-syn multiplication across multiple biological scales. This model integrates genetic, cellular, molecular, and systems-level mechanisms that collectively determine α-syn burden and pathogenic activity.

At the genetic level, *SNCA* gene dosage is a major determinant of α-syn expression, as demonstrated by copy-number variants associated with familial PD caused by *SNCA* duplication and triplication (3–5). At the cellular level, protein homeostatic mechanisms, particularly the autophagy–lysosome system, regulate α-syn turnover, and their impairment promotes progressive intracellular accumulation of α-syn species (6, 7). At the molecular level, elevated α-syn concentration alters its conformational landscape and may promote the formation and stabilization of neurotoxic oligomeric assemblies (8). *Under physiological conditions.*

At the systems level, increased α-syn production, impaired clearance, and enhanced formation of pathogenic species may collectively contribute to progressive dysfunction of vulnerable neuronal populations, particularly within the nigrostriatal pathway. These processes may subsequently facilitate the network-level propagation of pathology, consistent with the characteristic progression of PD (9–11).

Together, these interconnected processes support a unified concept of α-syn multiplication, in which disease severity and progression are shaped by the balance among α-syn production, clearance, and conformational dynamics.

### 2.5 Convergence of pathogenic mechanisms

The evidence discussed above suggests that diverse genetic, molecular, and cellular abnormalities in PD may converge on a shared pathogenic axis characterized by increased α-syn burden and impaired proteostatic control (2–8, 11). Although familial and sporadic PD may arise from distinct initiating factors, both may involve an imbalance between α-syn production and clearance capacity (3–7).

**Figure 3:**
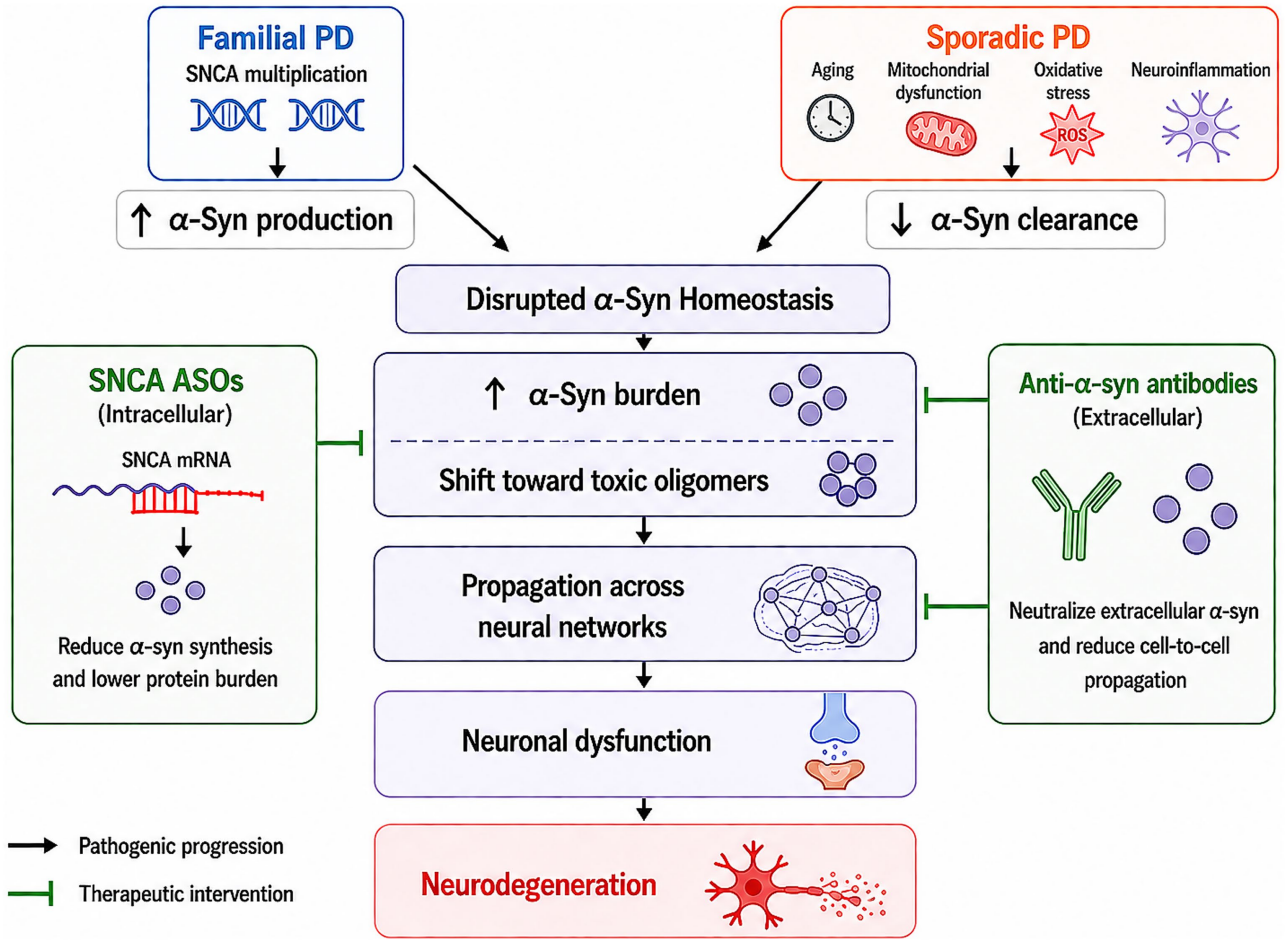
Convergence of pathogenic mechanisms and therapeutic intervention points in Parkinson’s disease. This schematic illustrates the convergence of familial and sporadic Parkinson’s disease (PD) on a shared pathogenic pathway characterized by increased α-synuclein (α-syn) burden. In familial PD, *SNCA* copy-number multiplication directly increases α-syn production through a gene-dosage effect. In sporadic PD, aging, mitochondrial dysfunction, oxidative stress, and neuroinflammation may impair α-syn clearance and disrupt α-syn homeostasis. Increased α-syn burden may promote the formation of oligomeric species with pathogenic potential, contributing to neuronal dysfunction, network-level propagation, and progressive neurodegeneration. The figure also highlights major candidate disease-modifying strategies. Anti-α-syn monoclonal antibodies aim to limit extracellular α-syn propagation, whereas *SNCA*-targeting antisense oligonucleotides (ASOs) seek to reduce intracellular α-syn synthesis and overall protein burden. Together, these mechanisms illustrate the proposed concept of PD as a disorder characterized by α-synuclein multiplication.

In hereditary PD associated with *SNCA* copy-number multiplication, increased gene dosage directly elevates intracellular α-syn levels and constitutes an important upstream source of protein burden (3–5). In sporadic PD, age-related proteostatic decline, mitochondrial dysfunction, oxidative stress, and neuroinflammation may reduce α-syn clearance capacity, thereby increasing the effective intracellular α-syn burden (2, 6, 7). Thus, distinct upstream triggers may converge on a common state of disrupted α-syn homeostasis (Figure 3).

As α-syn levels increase, the conformational distribution may shift toward oligomeric assemblies, some of which exhibit substantial pathogenic potential (8). This concentration-dependent shift provides a potential mechanistic link between protein abundance and neurotoxicity. Autophagy–lysosome dysfunction may further amplify this process by reducing degradative capacity and promoting the accumulation of pathogenic species (6, 7).

Elevated α-syn levels may facilitate the intercellular transmission of pathogenic species, thereby contributing to the propagation of pathology through neural networks (9–11). This suggests that α-syn multiplication may operate not only at the intracellular level but also at the systems level by extending pathology across neural networks over time.

Consistent with this framework, emerging disease-modifying strategies have targeted α-syn at both extracellular and intracellular levels. Passive immunotherapy with anti-α-syn monoclonal antibodies has been evaluated in clinical trials to neutralize extracellular α-syn species and limit cell-to-cell propagation (24, 25). In the phase II PASADENA trial of prasinezumab in early PD, clinical progression and biomarker outcomes were assessed, but the primary endpoints were not met (24). Similarly, the SPARK trial evaluated cinpanemab in early-stage PD but was terminated because no clinical benefit was observed (25).

In parallel, nucleic acid-based approaches, including antisense oligonucleotides (ASOs) targeting *SNCA*, are being developed to reduce α-syn synthesis at the transcript level (26, 27). These strategies aim to decrease intracellular α-syn production and thereby reduce protein burden upstream of aggregation and toxicity. Preclinical studies have shown that *SNCA* knockdown can reduce α-syn pathology and ameliorate neurodegenerative phenotypes, supporting the therapeutic rationale for lowering α-syn expression (26). Collectively, these interventions target complementary components of the α-syn multiplication framework by reducing extracellular propagation and intracellular α-syn production. These approaches are consistent with current concepts of disease-modifying therapy for PD (28, 29).

## 3 Discussion

This review proposes that PD can be interpreted through a framework of quantitative α-syn multiplication operating across genetic, cellular, molecular, and systems levels (2–11, 21, 22, 26, 27). This framework integrates established genetic evidence with mechanistic and experimental findings and may help unify processes implicated in both familial and sporadic PD (2–11, 21, 22, 26, 27).

A central component of this model is the gene-dosage effect observed in *SNCA* copy-number variants. *SNCA* duplication is associated with a relatively typical, later-onset parkinsonian phenotype, whereas *SNCA* triplication is associated with earlier onset, more rapid progression, and broader neurological involvement (3–5). This stepwise genotype–phenotype relationship supports the view that α-syn toxicity is sensitive to protein abundance and may be dose-dependent (3–5).

Experimental studies further suggest that the consequences of increased α-syn burden are modified by proteostatic capacity. In particular, disruption of autophagy in α-syn overexpression models accelerates neurodegeneration, indicating that intracellular clearance systems modulate α-syn toxicity (6, 7, 21). These findings highlight the interaction between α-syn production and degradation pathways in shaping neuronal vulnerability.

At the molecular level, α-syn occupies a dynamic conformational landscape comprising monomeric, oligomeric, and fibrillar states (8). Oligomeric species are increasingly implicated in neurotoxicity, and their formation is influenced by α-syn concentration (8). Thus, even modest increases in α-syn levels may shift the conformational distribution toward pathogenic assemblies, linking quantitative changes in protein burden with qualitative changes in toxicity.

Within this framework, *SNCA* copy-number multiplication and impaired proteostasis may converge on a shared pathogenic mechanism: a concentration-dependent shift in α-syn conformational distribution combined with reduced clearance capacity (3–8). This dual-hit model offers a potential explanation for disease heterogeneity and progression.

This model may also help bridge mechanisms implicated in familial and sporadic PD. Whereas *SNCA* copy-number multiplication directly increases α-syn production (3–5), sporadic disease may involve cumulative mitochondrial dysfunction, age-related proteostatic decline, and impaired autophagic activity (2, 6, 7, 21, 22). In both settings, a common downstream feature may be an imbalance between α-syn burden and cellular clearance capacity.

From a systems perspective, increased α-syn levels may facilitate the intercellular propagation of pathogenic species, thereby contributing to the characteristic spatiotemporal progression of PD pathology (9–11). This supports the hypothesis that α-syn multiplication increases intracellular toxicity and promotes the spread of pathology across neural circuits.

### 3.1 Therapeutic implications

This framework has several therapeutic implications. Rather than targeting aggregation alone, candidate disease-modifying strategies may need to reduce α-syn production, enhance autophagic and lysosomal function, and limit intercellular transmission (22, 26, 27). Such multilevel targeting may be needed to interrupt the proposed amplification loop that contributes to disease progression.

### 3.2 Limitations and future directions

Several limitations of the proposed framework should be acknowledged. The quantitative relationship between α-syn concentration and conformational-state distribution *in vivo* remains incompletely defined. The temporal sequence linking proteostatic failure, α-syn accumulation, and neurodegeneration is also not fully understood. In addition, the supporting evidence is heterogeneous across genetic studies, experimental models, neuropathological observations, and clinical investigations. Furthermore, the proposed framework may not apply uniformly across all PD subtypes, and an operational definition of the effective α-syn burden has yet to be established. Future studies integrating quantitative proteomics, longitudinal imaging, and refined *in vivo* models will be important for clarifying these dynamics. Longitudinal studies that quantify α-syn production, clearance, conformational state, and regional propagation within the same experimental models or patient populations will be needed to determine whether these processes constitute a measurable multiplication axis.

## 4 Conclusion

Accumulating evidence supports α-syn multiplication as an integrative framework for understanding PD beyond a protein-misfolding model alone. This model integrates gene-dosage effects, proteostatic regulation, conformational dynamics, and propagation mechanisms into a unified framework that may help connect familial and sporadic PD and identify multiple potential therapeutic entry points (2–11, 21, 22, 26, 27). Environmental factors, including pesticides and other neurotoxins, may further exacerbate these pathogenic processes by promoting α-syn accumulation, impairing proteostasis, and inducing mitochondrial dysfunction, thereby supporting the concept that genetic and environmental factors converge on a common α-syn–centered pathogenic pathway in PD. The model’s validity will depend on whether α-syn production, clearance, conformational redistribution, and network propagation can be measured as interdependent components of disease progression.
